# A População Brasileira Apresenta Prevalência de Fibrilação Atrial Semelhante à de Países com Rendas mais Altas, e Baixo Uso de Terapia Anticoagulante

**DOI:** 10.36660/abc.20210562

**Published:** 2021-09-01

**Affiliations:** 1 Faculdade de Medicina da Universidade de São Paulo Hospital das Clínicas Instituto do Coração São Paulo SP Brasil Faculdade de Medicina da Universidade de São Paulo, Hospital das Clínicas, Instituto do Coração – Unidade Clínica de Aterosclerose, São Paulo, SP – Brasil

**Keywords:** Fibrilação atrial, Epidemiologia, Prevalência, Fatores de Risco, Taxa de Sobrevida, Adulto Jovem, Pessoa de Meia Idade, Arritmias Cardíaca

A fibrilação atrial (FA) é a arritmia mais incidente e sua frequência tem aumentado, já que uma proporção maior de pessoas com idade acima dos 60 anos tem se tornado uma tendência mundial.

Há um aumento exponencial da FA com o avanço da idade a partir da faixa dos 50 – 59 anos, de 5 vezes na faixa dos 60 – 69 anos, de 7 vezes na faixa dos 70 – 79 anos, e de 9 vezes acima dos 80 anos de idade.^1^

Os homens apresentam uma incidência mais alta de FA.^1^^-^^3^

Uma grande pesquisa envolvendo veteranos do sexo masculino também demonstrou diferenças étnicas na prevalência da FA padronizada pela idade: 3% em hispânicos, 3,4% em negros, 3,6% em asiáticos, 5,2% em ilhéus do Pacífico, 5,4% em indígenas norte-americanos e 5,7% em brancos.^4^

Os outros fatores de risco da FA incluem sedentarismo, tabagismo, obesidade, diabetes mellitus, apneia obstrutiva do sono, hipertensão, consumo de álcool, doença cardíaca coronária e insuficiência cardíaca.^5^

Uma análise sistemática de estudos publicados desde 2015 mostrou que 30% dos acidentes vasculares cerebrais foram causados pela FA.^6^

Como observado nas considerações iniciais, a FA é uma doença potencialmente perigosa com grande impacto em invalidez, mortes e gastos com saúde.^7^ Esse último foi estudado em detalhes por Barros e Silva et al.^8^

A taxa de mortalidade brasileira por acidente vascular isquêmico padronizada por idade e suas complicações no ano de 2019 era 28,62/100.000 habitantes entre 35 e 74 de idade (37,10 em homens e 21,39 em mulheres) (calculada a partir de dados do DATASUS e do IBGE com o software Joinpoint).

Nesta edição, Santos et al.^9^ apresenta os resultados da prevalência de FA em um coorte abrangente no Brasil - o estudo ELSA-Brasil, envolvendo 15.105 funcionários públicos de seis capitais estaduais brasileiras (São Paulo, Belo Horizonte, Porto Alegre, Salvador, Rio de Janeiro e Vitoria).^10^

A frequência de FA de 2,5% determinada foi semelhante à encontrada em outros estudos internacionais,^1^^,^^11^ e à de um estudo retrospectivo do estado de Minas Gerais.^3^

As principais doenças associadas à FA foram a insuficiência cardíaca (RC 7,35), doença coronária (RC 5,11), febre reumática (RC 3,38), aumento da idade (RC 1,05 por ano), e hipertensão (RC 1,44). Os índices de terapia anticoagulante foram muito baixos, em 7,25% na condição basal. Embora essa baixa frequência de anticoagulação seja mais alta do que identificada na condição inicial do estudo KP–RHYTHM (0,7%), ainda é mais baixa do que após o diagnóstico nesse estudo (38%).^12^

Em conclusão, a carga da FA na população brasileira é semelhante à da população mundial e os baixos índices de anticoagulação autorrelatados pelos sujeitos, 85% dos casos, são uma combinação ruim para a saúde pública.
