# Supplementary material for: A População Brasileira Apresenta Prevalência de Fibrilação Atrial Semelhante à de Países com Rendas mais Altas, e Baixo Uso de Terapia Anticoagulante
Source: Arq Bras Cardiol. 2021 Sep 1;117(3):435–6. [Article in Portuguese] doi: 10.36660/abc.20210562 (PMC8462960; doi:10.36660/abc.20210562)
Supplement: Translation

# Translation: Brazilian Population Presents Prevalence of Atrial Fibrillation Similar to Higher Income Countries, and a Low Use of Anticoagulation Therapy

**Affiliations:** 1 São Paulo SP Brazil Faculdade de Medicina da Universidade de São Paulo, Hospital das Clínicas, Instituto do Coração – Unidade Clínica de Aterosclerose, São Paulo, SP – Brazil

**Keywords:** Atrial Fibrillation, Epidemiology, Prevalence, Risk Factors, Rate, Survival, Young Adult, Middle-Aged, Arrhythmias, Cardiac

Atrial fibrillation (AF) is the most incident arrhythmia and its frequency is increasing as a greater proportion of people over 60 years of age have been become a worldwide tendency.

There is an exponential increase of AF with the advancing of age from 50-59 years, of 5 fold in 60 – 69 years, of 7 fold in 70 – 79 years, and of 9 fold over 80 years.^1^

Men present a higher incidence of AF.^1^^-^^3^

A large survey in male veterans also showed ethnic differences in age-adjusted prevalence of AF: 3% in Hispanics, 3.4% in blacks, 3.6% in Asians, 5.2% in Pacific Islanders, 5.4% in Native Americans, and 5.7% in whites.^4^

Other risk factors for AF include a sedentary lifestyle, smoking, obesity, Diabetes Mellitus, obstructive sleep apnea, hypertension, alcohol consumption, coronary heart disease, and heart failure.^5^

A systematic review of studies published since 2015 showed that 30% of strokes were caused by AF.^6^

As observed from these initial considerations, AF is a potentially dangerous condition with a great impact on disability, death, and health costs.^7^ The latter has been studied extensively by Barros & Silva et al.^8^

Brazilian age-adjusted mortality rate by ischemic stroke and its complications in 2019 was 28.62/100,000 inhabitants between 35 to 74 years of age (37.10 in men and 21.39 in women) (calculated from DATASUS and IBGE data with Joinpoint software)

On this issue, Santos et al.^9^ presents the results of AF prevalence in an extensive Brazilian cohort – ELSA-Brasil: 15,105 civil servants from six Brazilian state capitals (São Paulo, Belo Horizonte, Porto Alegre, Salvador, Rio de Janeiro, and Vitoria).^10^

AF frequency of 2.5% was similar to other international studies^1^^,^^11^ as well as to a retrospective Brazilian study from the state of Minas Gerais.^3^

The main associated conditions with AF were heart failure (OR 7.35), coronary disease (OR 5.11), rheumatic fever (3.38), age increment (OR 1.05 per year), and hypertension (OR 1.44). The rate of anticoagulation therapy was very low, 7.25% in the basal condition. Although low, this anticoagulation frequency was higher than that found in the initial condition of KP-RHTYTHM (0.7%) but less than after diagnosis in that study (38%).^12^

In conclusion, the AF burden in the Brazilian population is similar to the global population and the poor anticoagulation rate in the self-reporting subjects, 85% of all cases, a bad consortium for public health.
